# From tradition to trait: stay-green alleles in Sudanese *Feterita* unlock pathways to climate-resilient sorghum

**DOI:** 10.3389/fpls.2025.1705070

**Published:** 2026-01-14

**Authors:** Mohammed Elsafy, Aisha Abdalhady Ahmed Abdalla, Shama Abdo, Alaa Ahmed, Fluturë Novakazi, Helena Persson Hovmalm, Eva Johansson, Rodomiro Ortiz, Mahbubjon Rahmatov, Tilal Abdelhalim

**Affiliations:** 1 Department of Plant Breeding, Swedish University of Agricultural Sciences (SLU), Lomma, Sweden; 2 College of Agricultural Studies, Sudan University of Science and Technology, Shambat, Khartoum North, Sudan; 3 Biotechnology and Biosafety Research Center, Agricultural Research Corporation, Shambat, Khartoum North, Sudan; 4 Department of Crop Health, Faculty for Agriculture, Civil and Environmental Engineering, University of Rostock, Rostock, Germany

**Keywords:** drought tolerance, genetic diversity, KASP markers, sorghum, stay-green alleles

## Abstract

**Introduction:**

Sorghum is a vital crop for food and nutritional security in drought-prone regions. However, the genetic potential of Sudanese *Feterita* landraces for stay-green (*Stg*) traits remains largely unexplored.

**Methods:**

This study evaluated 133 *Feterita* genotypes using an integrated approach combining field phenotyping with KASP genotyping targeting the *Stg3A* and *Stg3B* QTLs. The genotypes were assessed during the 2022 season at the Gezira Research Station using an augmented design, and 14 morphological and agronomic traits were recorded for each genotype.

**Results and Discussion:**

Substantial phenotypic variation was observed, with particularly high coefficients of variation for flowering stalks per plant (81.25%), senescence (49.09%), and grain number per panicle (43.33%). Genetic diversity analysis revealed moderate marker informativeness (GD = 0.407, I = 0.293, and PIC = 0.592). The AMOVA indicated weak population differentiation (*Fst* = 0.014), with 94% of the variation occurring within populations and substantial gene flow (Nm = 6.117). Pairwise *Fst* identified West Darfur and Blue Nile as genetically distinct, whereas Kordofan, White Nile, and Al-Gezira formed a cohesive cluster. Significant marker-trait associations were detected, including *snpSB0054* (SGR1) with plant height and flowering time, *snpSB0072* with panicle length, and *snpSB0101* (SGR3) with grain number and seed yield. These findings highlight *Feterita* as a valuable reservoir of functional *Stg* alleles that influence senescence dynamics and drought-related yield traits. This study provides the first targeted characterization of stay-green alleles in Sudanese *Feterita* and lays the foundation for exploiting this germplasm in marker-assisted breeding to develop climate-resilient sorghum varieties in the future.

## 1 Introduction

Sorghum (*Sorghum bicolor* L. Moench) is a vital cereal crop, particularly in arid and semi-arid regions, where drought tolerance is critical for sustaining its yield (Faye et al., 2022; Harris-Shultz et al., 2019; Tsehaye et al., 2024). In Sudan, sorghum, locally known as “*Aish*,” meaning life, is cultivated on over 10 million hectares, primarily in the rain-fed areas of the Central Clay Plain. Sorghum grain is a key ingredient in traditional Sudanese dishes, including leavened flatbread (*Kisra*), stiff porridge (*Aceda)*, boiled and soaked grains (*Balila*), thin porridge (*Nasha* and *Madida*), and non-alcoholic beverages (Abreh). In addition to its use as a food or drink, sorghum is a source of fodder, feed, and biofuel (Rao et al., 2019; Silva et al., 2022). Despite its importance, sorghum productivity in Sudan remains low, averaging less than 400 kg/ha, which is substantially below both the African (~ 1.2 t/ha) and global (~ 1.5–1.6 t/ha) averages (Elagib et al., 2019; Elhag and Zhang, 2018; FAOSTAT, 2023; Khalifa and Eltahir, 2023). This low yield is partly due to inadequate agronomic practices, such as insufficient fertilizer use and limited access to drought-tolerant seed varieties. Therefore, improving drought tolerance in sorghum is a key goal for plant breeders to reduce yield losses in moisture-stressed environments (Mundia et al., 2019).

The stay-green (*Stg*) trait, initially identified in the drought-tolerant donor B35, is controlled by four major quantitative trait loci (QTL), including *Stg1*, *Stg2*, *Stg3*, and *Stg4* (Ngugi et al., 2013). Fine mapping of these QTL has identified candidate genes that contribute to delayed senescence, a crucial factor in drought tolerance (Kiranmayee et al., 2020). Recent integrative genomic studies have shown that several stay-green–associated loci co-localize with QTL controlling plant architecture and biomass yield, indicating the shared genetic regulation of drought adaptation and canopy development in sorghum (Singh et al., 2025). These loci also influence key hormonal pathways, particularly abscisic acid (ABA) and cytokinins, which coordinate drought perception, stomatal behavior, and leaf senescence (Jan et al., 2019). Increased ABA sensitivity enhances early drought signaling and osmotic adjustment, whereas sustained cytokinin levels delay chlorophyll degradation and preserve sink strength. Together with improved reactive oxygen species (ROS) scavenging, this hormonal and oxidative balance provides a physiological basis for the stay-green phenotype and its resilience to post-flowering drought stress (Abbas et al., 2025).

Incorporating these QTL into breeding programs will enable the development of sorghum varieties with enhanced drought resistance, positioning the *Stg* trait as a central target for improving drought tolerance in sorghum. Recent advances in Kompetitive Allele-Specific PCR (KASP) technology have enabled cost-effective single-nucleotide polymorphism (SNP) genotyping, making marker-assisted selection (MAS) a valuable tool in sorghum breeding. KASP has been applied to sorghum for drought tolerance, including the validation of favorable Stg alleles in BC2F3 populations in Tanzania (Semagn et al., 2014; Sejake et al., 2021; Mwamahonje et al., 2021). These studies demonstrate the potential of KASP to accelerate the introgression of Stg QTL into farmer-preferred cultivars. According to Kiranmayee et al. (2020), two QTLs, *Stg3A* and *Stg3B*, associated with leaf greenness under post-drought stress in sorghum, were mapped to the 56–72 Mbp region of chromosome SBI-02. These QTL are related to transpiration efficiency and response to vapor pressure deficit (Ayalew et al., 2018; Deshpande et al., 2016).

*Feterita* is a unique sorghum breeding pool found only in Sudan, originating from the natural cross-hybridization between the *Caudatum* and *Guinea* races in the Nuba Mountains (Dirar, 1994). Farmers favor *Feterita* due to its adaptability to harsh environments; however, it remains underutilized in sorghum breeding programs for drought tolerance, with limited research on its *Stg* traits. Despite its cultural, nutritional, and agronomic relevance, the adoption and improvement of Feterita face several bottlenecks. These include the absence of structured seed systems for dissemination, susceptibility of traditional landraces to storage pests, inconsistent grain quality demanded by different markets, and limited breeding investments targeting their unique attributes. In addition, many Feterita accessions exhibit tall stature, which can reduce the adoption of mechanized rainfed systems by farmers. These constraints have slowed the integration of Feterita into national breeding pipelines, leaving its adaptive potential largely unexploited. Therefore, the present study was designed to examine the *Feterita* gene pool for Stg alleles using KASP markers linked to Stg3A and Stg3B QTL from donor parents B35 and S35. To our knowledge, this is the first study to investigate Sudanese *Feterita* as a source of drought tolerance based on *Stg*, offering novel perspectives on the development of drought-resilient cultivars.

## 2 Materials and methods

### 2.1 Plant material

In this study, we analyzed 133 sorghum landraces from *the Feterita* breeding pool. Sudanese farmers widely prefer these landraces. The genotypes were obtained from the Plant Genetic Resources Conservation and Research Center (APGRC) of the Agricultural Research Corporation (ARC) in Sudan. Detailed passport data, including accession numbers, variety names, collection and acquisition dates, characterization years, collection sites with geographic coordinates (latitude, longitude, altitude), and corresponding regions/states, are presented in **Supplementary Table 1**.

### 2.2 Phenotypic data

Phenotypic data were collected from field evaluations of the genotypes conducted at the Research Farms of the Agricultural Plant Genetic Resources Conservation and Research Center (APGRC) at Gezira Research Station in Sudan. The genotypes were evaluated during the 2022 growing season under field conditions using an augmented design with one-row plots (5 m each), spaced 30 cm between plants and 80 cm between ridges. The traits included ten quantitative characteristics: plant height (cm), number of leaves, days to 50% flowering, number of flowering stalks per plant, number of tillers per plant, inflorescence exertion, inflorescence length (cm), inflorescence width (cm), 100-seed weight (g), and grain number per panicle, and four qualitative traits: leaf midrib color, overall plant aspect, synchrony of flowering, and intensity of fully developed leaf color (senescence), according to the descriptor list provided by the APGRC (**Supplementary Table 2**). The leaf midrib color was visually scored on a 1–3 scale (1 = green, 2 = light purple, 3 = deep purple) following the sorghum descriptor guidelines of IBPGR/ICRISAT (1993). Scoring was performed at the soft-dough stage on the third fully expanded leaf using the Munsell Plant Tissue Color Chart to ensure consistency between observers. The recorded values ranged from 1 to 3 (mean = 1.82), indicating a high variability (CV = 45.6%). In designing the study, particular emphasis was placed on generating a broad and high-resolution phenotypic dataset, so that the limited but functionally targeted KASP marker set could be interpreted in the context of multi-trait variation related to stay-green, flowering behavior, and yield component traits.

### 2.3 Genotyping

Five seeds representing each genotype were randomly selected and planted in plastic nursery trays in a greenhouse of the Department of Plant Breeding at the Swedish University of Agricultural Sciences (SLU), Alnarp, Sweden. Two weeks after germination, young and healthy leaves from two to three individuals per genotype were collected as described by Pipan et al. (2017). The harvested leaf tissues were placed in sterile polypropylene deep-well plates (96-well), sealed with Parafilm to maintain moisture content and prevent airborne contamination, and dried in a freeze dryer under the following conditions: temperature, –10 to –70°C; pressure, 0.05–0.5 mbar; and duration, 48–72 h (Wolkers and Oldenhof, 2020).

Dried leaf samples were sent to Intertek-AgriTech (https://www.intertek.com/agriculture/agritech) in Sweden for genotyping. Ten KASP markers (*SnpSB0035*, *SnpSB0040*, *SnpSB0049*, *SnpSB0054*, *SnpSB0072*, *SnpSB0091*, *SnpSB0095*, *SnpSB0098*, *SnpSB0101*, and *SnpSB0103*), which were obtained from CGIAR (Mwamahonje et al., 2021) and were tightly associated with Stg3A and Stg3B QTLs, were used to screen Sudanese *Feterita* genotypes and trace both favorable and alternative alleles linked to the stay-green trait. KASP (Kompetitive Allele-Specific PCR) assays were performed according to the standard protocol provided by LGC. Each reaction included two allele-specific forward primers and one common reverse primer, with fluorescent reporters FAM and HEX to distinguish between alleles. PCR amplification and endpoint fluorescence detection were performed using a Roche LightCycler^®^ 480 platform, and genotype calling was performed using KlusterCaller™ software. Only samples with apparent bi-allelic clustering and a call rate of > 95% were retained for downstream analyses.

The number of SNPs (10 KASP markers) used in this study was modest compared with genome-wide SNP panels; however, these markers were deliberately selected because they were tightly linked to the Stg3A and Stg3B QTL and included variants with demonstrated functional roles in stay-green responses and drought tolerance. Detailed SNP marker information, including SNP ID, chromosome location, physical position (in Mb), favorable and alternative alleles, SNP/INDEL classification, and associated gene/QTL, is listed in **Table 1**.

**Table 1:** KASP markers linked to stay-green QTLs (Stg3A and Stg3B) in sorghum, showing chromosome assignment, physical position (Mbp), favorable and alternate alleles, SNP/INDEL classification, and associated gene/QTL annotation.

S. No.	SNP ID	Chromosome	Physical_Mbp	Favorable allele	Alternate allele	SNP/INDEL	Gene/QTL
1	*snpSB0035*	2	56112177	T	C	C/T	*Stg3A*
2	*snpSB0040*	2	59000770	C	T	C/T	*Stg3A*
3	*snpSB0049*	2	59821923	G	A	G/A	*Stg3A*
4	*snpSB0054*	2	60098184	G	A	G/A	*Stg3A*
5	*snpSB0072*	2	61811307	A	G	G/A	*Stg3A*
6	*snpSB0091*	2	67306935	C	A	A/C	*Stg3B*
7	*snpSB0095*	2	67710384	A	G	A/G	*Stg3B*
8	*snpSB0098*	2	69739036	G	C	C/G	*Stg3B*
9	*snpSB0101*	2	70523721	C	G	C/G	*Stg3B*
10	*snpSB0103*	2	71419274	G	C	C/G	*Stg3B*

### 2.4 Data analysis

Morphological data, comprising both qualitative and quantitative traits, were obtained from the passport and characterization records of the Agricultural Plant Genetic Resources Conservation and Research Center (APGRC) in Sudan. These data were generated from field evaluations conducted under an augmented design, in which each genotype was planted in a single-row plot (5 m long). For each plot, measurements were collected from five randomly selected plants and averaged to obtain plot-based means for subsequent analysis. The dataset was subjected to descriptive statistical analyses, including minimum and maximum values, means, standard deviations, variances, and coefficients of variation, to summarize the phenotypic variability among the accessions.

The Gezira region is characterized by semi-arid conditions, with mean annual temperatures ranging from 29°C to 33°C and average yearly rainfall of approximately 170–180 mm. The soil is predominantly heavy clay, typical of the Central Clay Plain, providing a representative environment for evaluating drought-responsive traits in Sudanese sorghum germplasms.

Genotypic data were analyzed using GenAlEx 6.5.2 software (Peakall and Smouse, 2006) to assess genetic diversity at both the locus and genotype levels. The parameters included the number of alleles (Na), effective number of alleles (Ne), Shannon’s information index (I), observed heterozygosity (Ho), expected heterozygosity (He), fixation index (F), and number of private alleles (No. PA). AMOVA was performed to partition genetic variation within and among accessions and across their geographical origins. Additional diversity parameters, including gene diversity, allele frequency distribution, and polymorphic information content (PIC), were computed using R software version 3.4.4 (https://www.r-project.org).

The genetic structures of the *Feterita* accessions were inferred using Bayesian clustering implemented in STRUCTURE 2.3.4 (Pritchard et al., 2000). The number of clusters (K) was tested from 1 to 10, and the optimal K was identified using STRUCTURE HARVESTER (Earl and VonHoldt, 2012). The results were visualized as bar plots using Clumpak beta (Raj et al., 2014).

## 3 Results

### 3.1 Phenotype data

Morphological and agronomic evaluations of the *Feterita* sorghum genotypes revealed broad and striking phenotypic variations across the 14 recorded traits (**Table 2**, **Supplementary**). Plant height displayed the widest range, stretching from 56 to 380 cm, with a mean of 202.5 cm, a standard deviation of 75.2 cm, and the highest coefficient of variation (CV) among all traits (37.25%). Senescence, however, proved even more variable in relation to its scale, ranging from 1 to 9, with a mean of 4.40 (SD = 2.16; CV = 49.1%). The values for leaf midrib color ranged from 1 to 3 (mean = 1.82), with high variability (CV = 45.6%) (**Table 2**).

**Table 2:** Provides a summary of the descriptive statistics for the 14 quantitative and qualitative traits of the *Feterita* genotypes under study. Results are based on passport data for the genotypes from previous phenotyping, made available by APGRC.

Trait	Min	Max	Mean	Variance (n)	SD (n)	CV (%)
Plant height (cm)	56	380	202.54	5,661.34	75.24	37.15
Leaf midrib color	1	3	1.82	0.69	0.83	45.60
Number of leaves	7	19	14.37	8.77	2.96	20.60
Senescence	1	9	4.40	4.68	2.16	49.09
Overall plant aspect	3	7	5.34	1.52	1.23	23.03
Days to 50% flowering	46	146	84.23	491.96	22.18	26.33
Number of flowering stalks/plants	0	7	1.28	1.08	1.04	81.25
Number of tillers/plants	0	3	1.47	0.36	0.60	40.82
Synchrony of flowering	0	1	1.03	9.29	0.03	2.91
Inflorescence exertion	1	4	1.90	0.73	0.85	44.74
Inflorescence length (cm)	13	38	22.53	22.89	4.78	21.22
Inflorescence width (cm)	3	10	5.63	2.29	1.51	26.82
100-seed weight (g)	1.4	4.02	2.62	0.43	0.66	25.19
Grain number/panicle	226	3548	1.54	444.47	666.68	43.33

Min, minimum, Ma, maximum, SD, standard deviation, CV, coefficient of variation.

The number of flowering stalks per plant showed the greatest relative variability of all traits (CV = 81.25%), ranging from 0 to 7, with a mean value of 1.28. Inflorescence exertion ranged from 1 to 4 (mean = 1.90; CV = 44.74%), whereas grain number per panicle exhibited substantial differences, varying from 226 to 3,548 grains, with a mean of 1,538.49 (CV = 43.33%). The number of tillers per plant also varied considerably, ranging from 0 to 3 (mean = 1.47, CV = 40.82) (**Table 2**).

Regarding flowering traits, days to 50% flowering extended from 46 to 146 days (mean = 84.23; CV = 26.33%), whereas synchrony of flowering showed the least variation, with values confined to 0–1, a mean of 1.03, and a CV of only 2.91%, highlighting the strong synchrony among the genotypes (**Table 2**).

Inflorescence traits revealed moderate variability, with inflorescence width ranging from 3 to 10 cm (mean = 5.63 cm; CV = 26.82%) and inflorescence length ranging from 13 to 38 cm (mean = 22.53 cm; CV = 21.22%). The number of leaves per plant ranged from 7 to 19, with a mean of 14.37 (CV = 20.60) (**Table 2**).

Concerning yield-related traits, 100-seed weight varied between 1.4 and 4.02 g (mean = 2.62 g; coefficient of variation [CV] = 25.19%), whereas the overall plant aspect was more consistent, ranging from 3 to 7 (mean = 5.34; CV = 23.03%) (**Table 2**).

### 3.2 Genotyping results

Analysis of ten SNP markers across 133 *Feterita* sorghum genotypes revealed a distinct pattern in the distribution of favorable and heterozygous alleles (**Table 3**). Among these, *snpSB0101* (linked to the stay-green gene SGR3) showed the highest frequency of favorable alleles (n=131), followed by *snpSB0040* (n=115), which is associated with programmed cell death (PCD) triggering. In contrast, snpSB0098 (aspartic proteases mediated by salicylic acid [SA] signaling) showed no favorable alleles in the tested genotypes.

**Table 3:** Favorable alleles (FA) and heterozygous alleles (HA) were identified in 133 Sudanese *Feterita* sorghum genotypes using SNP markers associated with stay-green QTL (Stg3A and Stg3B), including their physical locations and corresponding functional traits.

SNP marker	FA	No. of FA	HA	No. of HA	QTL	Physical location	Functional trait
*snpSB0035*	TT	3	TC	2	*Stg3A*	56112177	Grain type
*snpSB0040*	CC	115	CT	4	*Stg3A*	59000770	PCD triggering
*snpSB0049*	GG	79	GA	16	*Stg3A*	59821923	N2 mobilization
*snpSB0054*	GG	30	GA	15	*Stg3A*	60098184	SGR1
*snpSB0072*	AA	33	AG	17	*Stg3A*	61811307	*APETALA2* and *EREBPs*
*snpSB0091*	CC	60	CA	12	*Stg3B*	67306935	Panicle compactness
*snpSB0095*	AA	75	AG	13	*Stg3B*	67710384	Salt responsive
*snpSB0098*	GG	0	GC	1	*Stg3B*	69739036	Aspartic proteases through SA
*snpSB0101*	CC	131	CG	0	*Stg3B*	70523721	SGR3
*snpSB0103*	GG	10	GC	5	*Stg3B*	71419274	SGR3

SNP, single nucleotide polymorphism, FA, favorable allele, HA, heterozygous alleles, QTL, quantitative trait loci, *Stg*: stay green gene. Grain type refers to loci associated with kernel morphology and end-use characteristics; PCD triggering denotes programmed cell death involved in senescence and stress regulation; N_2_ mobilization relates to nitrogen remobilization during grain filling; SGR1 and SGR3 are Stay-Green genes controlling chlorophyll degradation and delayed senescence; APETALA2 and EREBPs are transcription factors linked to drought and stress signaling; panicle compactness describes loci affecting panicle architecture and grain set; The term “salt-responsive” refers to loci that confer tolerance to salinity stress, while aspartic proteases, regulated by salicylic acid signaling, represent protease enzymes involved in stress and defense responses.

The markers were distributed across two key QTL regions: Stg3A (*snpSB0035*, *snpSB0040*, *snpSB0049*, *snpSB0054*, *snpSB0072*) and Stg3B (*snpSB0091*, *snpSB0095*, *snpSB0098*, *snpSB0101*, *snpSB010*3), spanning physical positions from 56.1 Mbp (*snpSB0035*) to 71.4 Mbp (*snpSB0103*).

Heterozygous alleles were detected for all markers, except for *snpSB0101*, with frequencies ranging from 1 (*snpSB0098*) to 17 (*snpSB0072*). SNP markers were linked to diverse functional traits, including grain morphology, programmed cell death, nitrogen remobilization, stay-green responses, panicle compactness, and salt responsiveness (**Table 3**).

*SnpSB0035* showed the lowest frequency of favorable alleles (n = 3) while maintaining a modest heterozygous frequency (n = 2). The core stay-green variants were represented by three markers: *snpSB0054*, *snpSB0101*, and *snpSB0103* (**Table 3**).

### 3.3 Genetic diversity results

Genetic diversity parameters revealed varying levels of polymorphism across the *Feterita* genotype collection (**Table 4**). The major allele frequencies (MAF) ranged from 0.015 (SNP35) to 0.127 (SNP72), with a mean of 0.079. The number of alleles (Na) varied from 1.000 (SNP40) to 1.857 (SNP54), whereas the effective number of alleles (Ne) ranged from 0.867 (SNP40) to 1.582 (SNP49), with a mean value of 1.259. Shannon’s information index (I) was highest at SNP95 (0.484) and lowest at SNP35 (0.033) (**Table 4**).

**Table 4:** Genetic diversity parameters of eight KASP markers used for molecular characterization of Sudanese *Feterita* sorghum genotypes.

Loci	MAF	Na	Ne	I	GD	He	Fis	Fst	Nm	PIC
SNP40	0.03	1.000	0.867	0.152	0.240	0.099	0.288	0.858	0.041	1,759
SNP54	0.112	1.857	1.391	0.399	0.505	0.254	0.209	0.202	0.990	1.494
SNP72	0.127	1.714	1.332	0.340	0.532	0.217	-0.272	0.349	0.466	1.467
SNP35	0.015	1.286	1.014	0.033	0.073	0.014	-0.169	0.018	14.000	1.926
SNP49	0.12	1.714	1.582	0.455	0.551	0.318	-0.275	0.169	1.227	1.448
SNP91	0.09	1.714	1.355	0.340	0.577	0.219	0.356	0.197	1.021	1.422
SNP95	0.098	1.714	1.430	0.484	0.538	0.328	0.268	0.433	0.328	1.461
SNP103	0.038	1.429	1.100	0.140	0.241	0.079	-0.350	0.082	2.793	1.758
Mean	0.079	1.554	1.259	0.293	0.407	0.191	0.007	0.288	2.608	1.592
Max	0.127	1.857	1.582	0.484	0.577	0.328	0.356	0.858	14.000	1.926
Min	0.015	1.000	0.867	0.033	0.073	0.014	-0.350	0.018	0.041	1.422

MAF, major allele frequency; Na, number of alleles; Ne, effective number of alleles; I, Shannon’s information index; GD, gene diversity; He, expected heterozygosity; F, inbreeding coefficient; Fis, fixation index within subpopulations; Fst: fixation index measuring genetic differentiation among subpopulations; Nm, gene flow estimated from Fst = 0.25 (1 – Fst)/Fst; PIC, polymorphism information content.

The gene diversity (GD) ranged from 0.073 (SNP35) to 0.577 (SNP91), with an average of 0.407. The expected heterozygosity (He) was lowest at SNP35 (0.014) and highest at SNP95 (0.328), with an average He of 0.191. The fixation index (Fis) values fluctuated widely, from -0.350 (SNP103) to 0.356 (SNP91), with an overall mean near zero (0.007), indicating minimal inbreeding within the populations. The genetic differentiation (Fst) ranged from 0.018 (SNP35) to 0.858 (SNP40), with a mean of 0.288, indicating both an excess and a deficit of heterozygosity across loci. Gene flow estimates (Nm) varied considerably, from 0.041 (SNP40) to 14.000 (SNP35), with an average of 2.608. The polymorphism information content (PIC) ranged from 1.422 (SNP91) to 1.926 (SNP35), with a mean of 1.592, indicating that the markers were moderately to highly informative for assessing genetic diversity in the population (**Table 4**).

### 3.4 Genetic diversity patterns within and among populations

Analysis of the genetic diversity among the 131 *Feterita* genotypes collected from seven states (populations) (**Table 5**, **Supplementary Table 1**) revealed substantial variation in the sample size and genetic parameters. The He ranged from 0.579 in West Darfur to 0.788 in White Nile, with a mean value of 0.740. Ho followed a similar trend, ranging from 0.608 (West Darfur) to 0.771 (Northern State), with an average of 0.705. Na varied between 2.88 (West Darfur) and 6.75 (Kordofan), whereas the effective number of alleles (Ne) ranged from 2.47 (West Darfur) to 4.74 (White Nile), with a mean value of 4.07 (**Table 5**).

**Table 5:** Sample size (N) and genetic diversity estimates among seven *Feterita* populations.

Population	N	Na	Ne	I	Ho	He	Fst	NPA	No. LC. A (≤50%)	PPL%
West Darfur	5	2.88	2.47	0.95	0.608	0.579	-0.009	0.00	0.000	100.00%
Kordofan	41	6.75	4.07	1.57	0.649	0.754	0.136	0.75	0.125	100.00%
White Nile	42	6.00	4.74	1.62	0.723	0.788	0.083	0.00	0.250	100.00%
Sinnar	16	5.75	4.63	1.62	0.719	0.783	0.082	0.00	0.250	100.00%
Blue Nile	5	4.88	4.08	1.48	0.750	0.750	0.001	0.00	0.125	100.00%
Northern State	6	5.25	4.40	1.55	0.771	0.771	-0.003	0.00	0.250	100.00%
Al-Gezira	18	5.00	4.13	1.49	0.712	0.757	0.061	0.00	0.000	100.00%
Mean	19	5.21	4.07	1.47	0.705	0.740	0.050	0.11	0.143	100.00%

N, number of samples, Na, number of alleles, Ne, effective number of alleles, I, Shannon’s information index, Ho, observed heterozygosity, He, expected heterozygosity, Fst, fixation index, NPA, number of private alleles, No. LC. A (≤50%): number of locally common alleles (Freq. ≥ 5%) found in 50% or fewer populations, PPL%, percentage of polymorphic loci.

Shannon’s information index (I) was lowest in West Darfur (0.95) and highest in White Nile and Sinnar (1.62), with a mean value of 1.47. The fixation index (Fst) values ranged from -0.009 (West Darfur) to 0.136 (Kordofan), with a mean of 0.050, indicating low levels of inbreeding in the studied populations. In this study, private alleles were detected only in the Kordofan population (0.75). The number of locally common alleles found in 50% or fewer populations ranged from 0.000 to 0.250, with a mean of 0.143. All populations showed 100% polymorphic loci, indicating complete genetic polymorphism across the surveyed markers (**Table 5**).

Differences within the populations accounted for 94% of the genetic variation in the *Feterita* sorghum populations, as determined by analysis of molecular variance (AMOVA). In contrast, only 6% of the variation was explained by differences between populations (**Table 6**). Among-population variation was represented by a sum of squares of 29.088 with 6 degrees of freedom, a mean square of 4.848, and an estimated variance of 0.097. In contrast, within-population variation showed a sum of squares of 404.430 across 259 df, with a mean square of 1.562 and an estimated variance of 1.562.

**Table 6:** Analysis of molecular variance (AMOVA) showing the partitioning of genetic variation within and among populations.

Source	df	SS	MS	Est. Var.	%	P-value	Fst
Among populations	6	29.088	4.848	0.097	6%	0.002	0.014
Within populations	259	404.430	1.562	1.562	94%	0.001	
Total	265	433.519		1.658	100%	0.001	
Nm (haploid) populations	6.117						

df: degrees of freedom, SS: sum of squares, MS: mean square, Est. Var.: estimate of variance, %: percentage of variation, P-value: probability, The P(rand ≥ data) value for PhiPT is determined by standard permutation across the entire data set.

The total sum of squares was 433.519 across 265 degrees of freedom, with a total estimated variance of 1.658. The fixation index (Fst = 0.014) indicated low but significant genetic differentiation among populations (P < 0.01). The number of migrants per generation (Nm = 6.117) suggests extensive gene flow among populations (**Table 6**).

Pairwise genetic differentiation (Fst) among the seven geographic regions revealed varying levels of population structure (**Table 7**). The highest differentiation was observed between West Darfur and Northern State (Fst = 0.528), West Darfur and Blue Nile (Fst = 0.467), and West Darfur and Al-Gezira (Fst = 0.424), confirming West Darfur as the most differentiated population, with Fst values ranging from 0.247 to 0.528. The Sinnar population showed moderate differentiation from other regions, with Fst values ranging from 0.093 (*vs*. Kordofan) to 0.345 (West Darfur). Kordofan, White Nile, and Al-Gezira appeared to be more closely related, with low Fst values (0.020–0.050). The Blue Nile and Northern State populations had a mean FIS of 0.305 (**Table 7**).

**Table 7:** Estimate of population genetic differentiation measured by Fst among different geographic regions.

State	West Darfur	Kordofan	White Nile	Sinnar	Blue Nile	Northern State	Al-Gezira
West Darfur	–						
Kordofan	0.247	–					
White Nile	0.300	0.020	–				
Sinnar	0.345	0.093	0.101	–			
Blue Nile	0.467	0.172	0.160	0.244	–		
Northern State	0.528	0.124	0.130	0.272	0.305	–	
Al-Gezira	0.424	0.031	0.050	0.165	0.258	0.179	–

### 3.5 Geographical distribution and population structure

**Figure 1:**
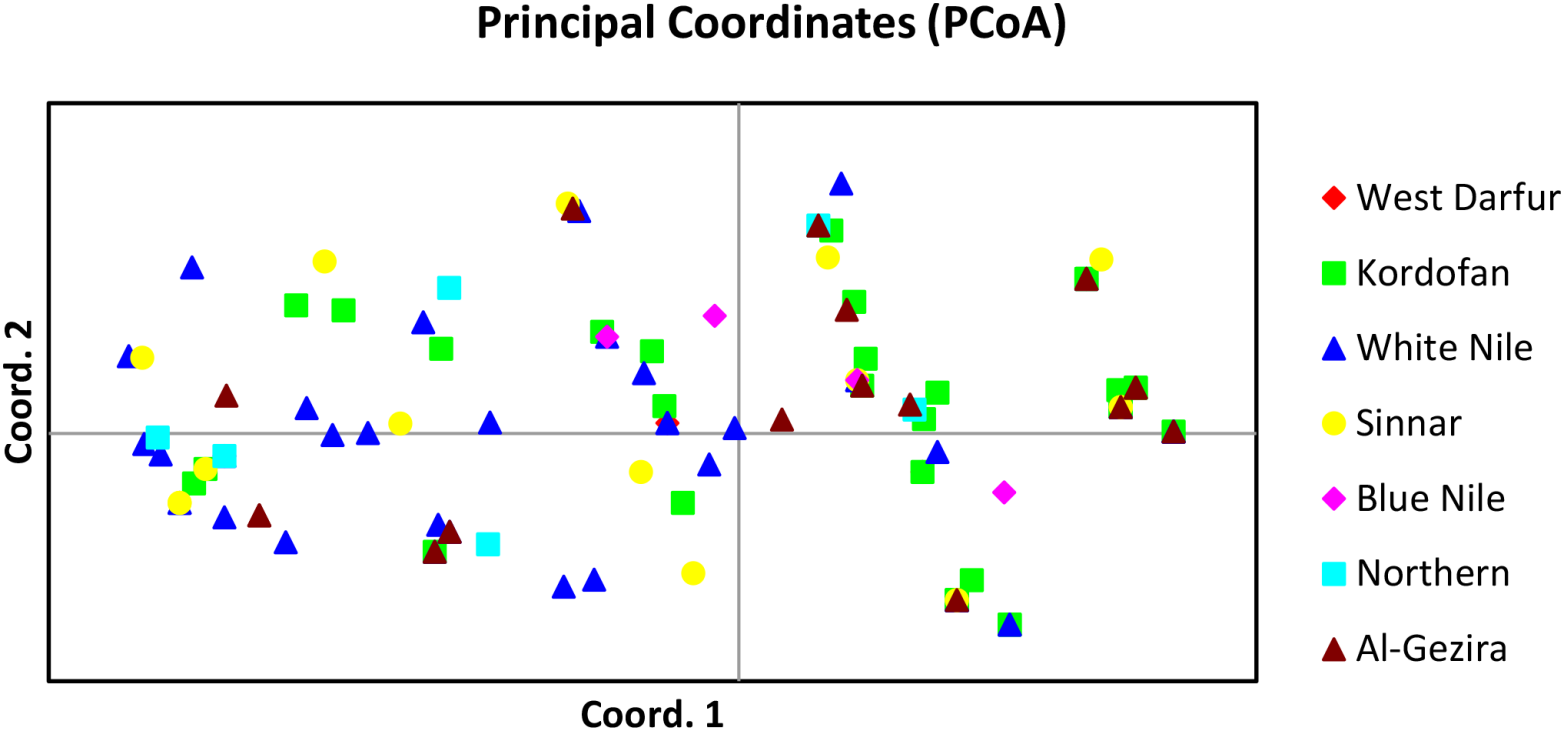
Principal coordinate analysis of 133 sorghum genotypes using eight KASP markers. Samples coded with the same symbols and colors belong to the same population. The percentages of variation explained 50.87% of the total variations, with the first three axes (1, 2, and 3) explaining 25.64%, 13.24%, and 11.99%, respectively.

**Figure 2:**
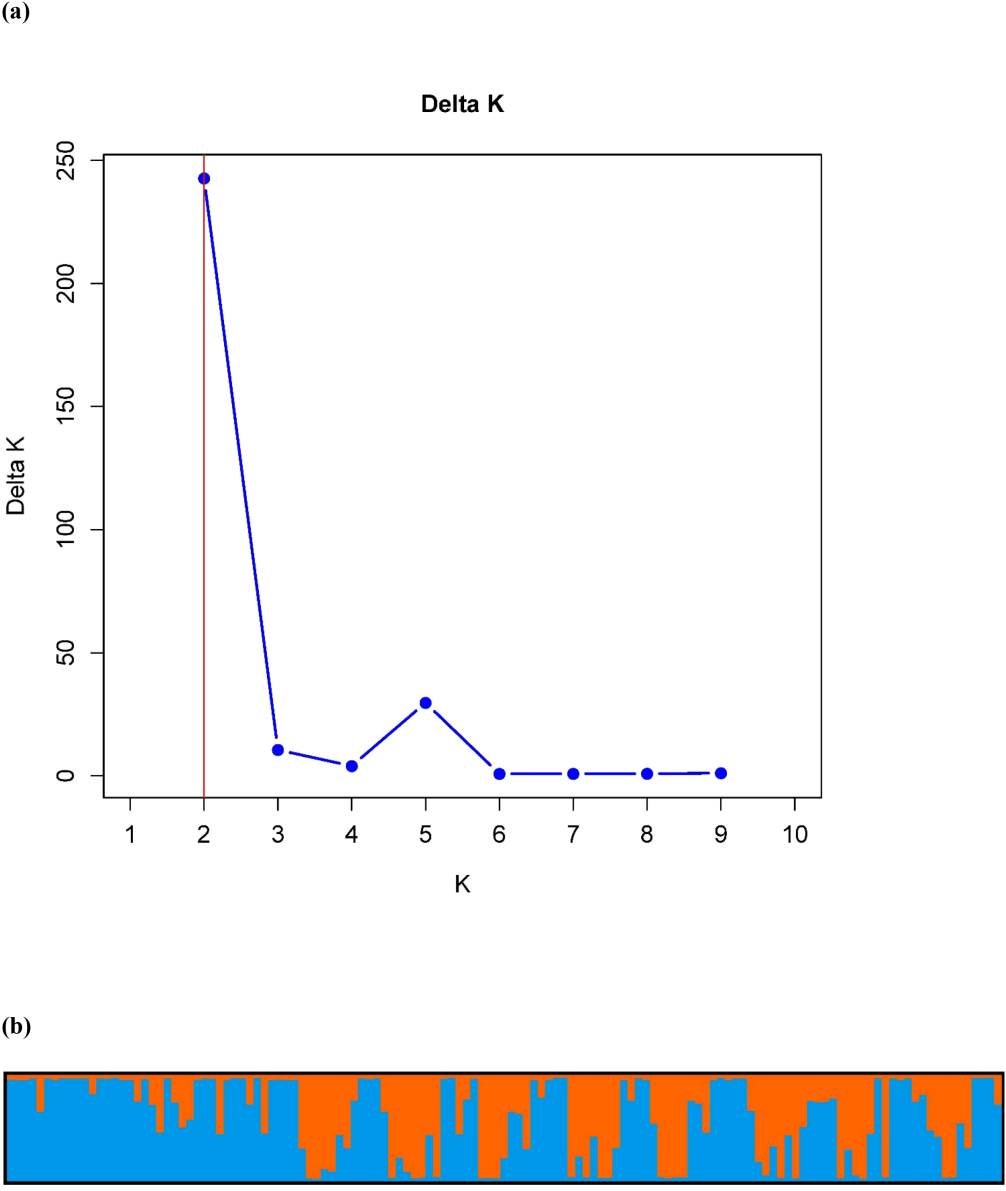
Population structure of 133 *Feterita* genotypes representing seven populations in Sudan. **(a)** Best delta K value estimated using the Evanno et al. (2005) method, and **(b)** Bayesian model-based estimated population structure for K = 2 according to geographical location. Different colors (blue and orange) represent genetic groups or subpopulations designated by Structure Harvester.

Principal coordinate analysis (PCoA) was conducted on 133 *Feterita* sorghum genotypes using eight KASP markers to assess genetic relationships and population structure (**Figures 1**, **2**). The first three axes explained 50.87% of the total genetic variation, with individual contributions of 25.64%, 13.24%, and 11.99% for axes 1, 2, and 3, respectively. The PCoA plot showed substantial intermixing of genotypes across all seven populations, with individuals from West Darfur, Kordofan, White Nile, Sinnar, Blue Nile, Northern State, and Al-Gezira scattered throughout coordinate space. The overlapping distribution of genotypes suggests a shared genetic background and potential gene flow among populations. The scattered arrangement of samples further reflects a continuum of gene variation rather than discrete, population-specific clusters, indicating that geographical origin does not strictly determine the genetic relationships among *Feterita* genotypes (**Figure 1**).

Population structure analysis identified two main genetic clusters (K = 2) among the 133 *Feterita* genotypes (**Figure 2A**). The delta K value peaked sharply at K = 2 (≈240), whereas the values for K = 3–10 were approximately zero. STRUCTURE plots (**Figure 2B**) showed that both clusters were represented across all seven geographical populations, with extensive admixture indicated by intermediate membership coefficients and mixed color patterns. No clear correspondence was observed between the genetic clusters and their geographical origins. The two clusters (K = 2) identified by STRUCTURE likely represent broad genetic differentiation corresponding to distinct ancestral lineages within the *Feterita* gene pool rather than discrete regional populations. This interpretation is supported by the principal coordinate analysis (PCoA) (**Figure 1**), where genotypes from both clusters were interspersed without clear geographic segregation. The substantial overlap observed in the PCoA plot (50.87% of the total variation explained by the first three axes) confirms extensive admixture and gene flow among regions, consistent with traditional farmer-mediated seed exchange across Sudan. Thus, while STRUCTURE detected two major genetic components, their biological significance likely reflects shared ancestry and historical recombination within an essentially continuous *Feterita* population.

### 3.6 Correlation analysis

**Figure 3:**
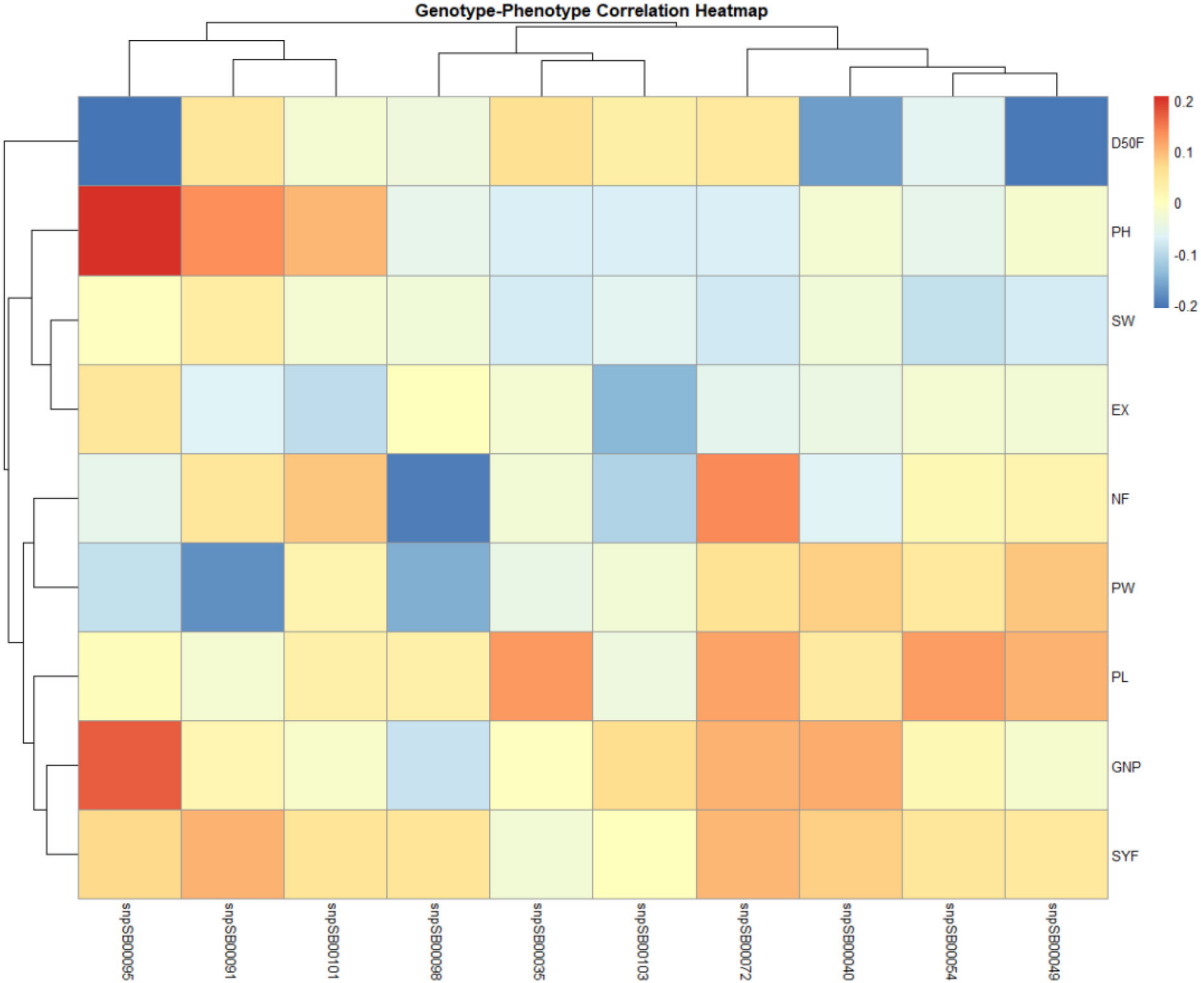
Correlation matrix of phenotypic traits and 10 SNP markers associated with stay-green traits in Sudanese *Feterita* genotypes. Marker and trait names have been enlarged to improve readability. Trait abbreviations: PH, plant height; D50F, days to 50% flowering; PL, panicle length; GNP, grain number per panicle; SYF, seed yield per plant; SW, 100-seed weight; EX, inflorescence exertion; NF, number of flowering stalks per plant; PW, panicle width.

Marker–trait associations (MTAs) were quantified using the coefficient of determination (R²) values to estimate the proportion of phenotypic variance explained by each marker (**Figure 3**). The significant associations included *snpSB0054* (SGR1) with plant height (R² = 0.28, P < 0.01) and days to 50% flowering (R² = 0.22, P < 0.05); *snpSB0072* with panicle length (R² = 0.19, P < 0.05); and *snpSB0101* (SGR3) with grain number per panicle (R² = 0.31, P < 0.01) and seed yield (R² = 0.27, P < 0.05). These values indicate moderate-to-strong associations, suggesting that the identified loci contribute to phenotypic variation in drought-resilient traits.

## 4 Discussion

Sorghum is a cornerstone crop for food and nutritional security in arid and semi-arid regions, where climate variability and recurrent droughts severely limit its productivity. In Sudan, *Feterita* represents a distinctive sorghum gene pool that has been cultivated for centuries and is valued for its adaptability and cultural significance in the region. However, its genetic potential remains largely untapped in modern breeding, particularly for drought-adaptive traits (Kimber et al., 2012). Among these, the stay-green (*Stg*) trait is essential, as it delays leaf senescence under post-flowering drought, sustains photosynthesis, and enhances yield stability in the remaining leaves. This study is the first to systematically mine *Feterita* germplasm for *Stg* alleles using molecular markers and phenotypic data, offering new insights into its value as a reservoir of adaptive variation for climate-resilient breeding programs.

The wide phenotypic variation observed among *Feterita* genotypes, particularly in plant height, senescence, and grain yield components, reflects their strong adaptive plasticity to semi-arid conditions. Such diversity provides a foundation for selecting contrasting parental lines for drought-resilient breeding, as phenotypic variability is crucial for capturing quantitative trait loci linked to the stay green phenotype (Harris-Shultz et al., 2019). The high coefficient of variation for senescence and flowering traits indicates differential drought-response mechanisms, consistent with the findings that delayed leaf senescence sustains photosynthesis and grain filling under moisture stress (Kiranmayee et al., 2020). These results confirm that phenotyping remains essential for validating molecular associations and designing effective multi-environment selection schemes.

Our findings indicate that the markers *snpSB0101* (SGR3) and *snpSB0054* (SGR1) exhibit high frequencies of favorable homozygous alleles, confirming their functional roles in regulating delayed leaf senescence under terminal drought conditions. These loci are directly linked to the stay-green (*Stg*) trait, which sustains photosynthetic capacity during the grain-filling period and enhances yield stability in moisture-limited environments. This result aligns with that of Mwamahonje et al. (2021), who identified these markers in BC_2_F_1_ populations of Sorghum. The high frequency of favorable alleles observed in our *Feterita* germplasm highlights their broad functional relevance across diverse millet genetic backgrounds. The Stg trait mediated by SGR1 and SGR3 is known to delay chlorophyll degradation and extend photosynthetic activity, thereby improving carbon assimilation and reproductive success under water-limited conditions (Borrell et al., 2014; Harris et al., 2007). Overall, the identification of these robust, functionally validated markers in Sudanese *Feterita* underscores their potential utility for marker-assisted backcrossing, providing a direct pathway for accelerating the development of drought-resistant sorghum cultivars.

A potential limitation of this study is that the field evaluation was conducted in a single growing season. While a single-season augmented trial is suitable for screening and ranking a large number of genotypes under uniform field conditions, genotype × environment interactions across years were not assessed, and multi-season testing will be required to confirm the stability and consistency of genotype performance. In addition, the restricted number of SNPs (10 KASP markers), which is lower than that in genome-wide diversity or association studies. However, this constraint is mitigated by the targeted selection of markers and depth of phenotyping. KASP markers were chosen because they are tightly linked to *Stg3A* and *Stg3B* and have been previously validated in stay-green and drought-tolerant backgrounds, thereby maximizing their functional relevance. In parallel, we recorded 14 morphological and agronomic traits, capturing wide variations in senescence, flowering time, plant architecture, and yield components under semi-arid field conditions. By integrating these multi-trait phenotypic data with Stg-linked markers, we identified robust marker–trait associations that elucidate how specific alleles contribute to drought-resilient performance in Feterita populations. Future work could complement this targeted framework with genome-wide SNP platforms (e.g., GBS or high-density arrays) and multi-environment trials to further dissect the genetic architecture of stay green and yield stability.

Our results revealed a high frequency of favorable and alternative alleles in the homozygous state across several stay-green (*Stg*)-associated loci, underscoring the genetic value of the Sudanese *Feterita* gene pool as a reservoir for drought tolerance. This allelic diversity likely reflects long-term evolutionary adaptation to semi-arid environments, where natural selection has favored genotypes capable of maintaining chlorophyll content and photosynthetic function under terminal drought stress. Comparable findings have been reported in other sorghum landraces and local varieties, which have been shown to harbor unique allelic combinations that contribute to stress resilience (Jordan et al., 2012; Mwamahonje et al., 2021). In our study, the presence of multiple favorable alleles within the *Feterita* genotypes underscores their untapped potential for improving sorghum. Such allelic richness provides opportunities for allele pyramiding in breeding programs, enabling the development of cultivars with enhanced and stable drought tolerance traits.

Our findings indicate that the genetic diversity of Sudanese *Feterita* sorghum genotypes, as revealed by KASP markers, is both moderate and highly informative for future breeding applications in Sudan. The mean gene diversity (0.407), Shannon’s information index (I = 0.293), and polymorphism information content (PIC = 1.592) collectively demonstrate the robustness of these markers in capturing allelic variation across populations. These values fall within the range reported in earlier sorghum diversity studies (Sejake et al., 2021), underscoring their reliability for genetic characterization.

Notably, the moderate-to-high marker informativeness observed in this study highlights substantial genetic diversity within the *Feterita* sorghum gene pool, which has been shaped by long-term adaptation to semi-arid, drought-prone environments in Sudan. Recent genomic studies of Ethiopian sorghum landraces have demonstrated pronounced population structure and high levels of allelic diversity associated with local adaptation and stress resilience (Enyew et al., 2022). The effective resolution of population structure using diagnostic marker systems is consistent with recent reports showing that cost-effective, high-throughput marker platforms such as KASP are well suited for diversity analysis and breeding-oriented applications in sorghum and other cereals (Baloch et al., 2023). Furthermore, the identification and utilization of stay-green–associated genomic regions remains a central strategy in contemporary sorghum breeding, given their documented role in post-flowering drought tolerance and yield stability under water-limited conditions (Velazco et al., 2019). Collectively, these findings reinforce the value of Feterita landraces as a strategic genetic resource for integrating farmer-preferred traits with modern climate-resilient sorghum improvement programs.

Our findings revealed that the fixation index (Fst = 0.007) was very low, indicating negligible population differentiation among the *Feterita* sorghum genotypes examined. This implies that most genetic variations persist within populations rather than across them. These results are consistent with those of our previous report on Sudanese sorghum germplasm, which highlighted the substantial allelic richness preserved through evolutionary processes (Ahmed et al., 2025). The observed diversity pattern can be explained by traditional Sudanese farming practices, where seed exchange across communities, limited formal selection pressure, and occasional outcrossing play central roles in maintaining broad genetic variation within local gene pools (Rabbi et al., 2010).

This study has two main implications for research and practice. First, the conservation of genetic diversity under traditional cultivation systems provides a broad adaptive base essential for resilience to environmental stressors. As highlighted by Altieri and Nicholls (2017), genetic diversity within landraces enhances the adaptive potential to drought, heat, and other abiotic stresses. Second, this reservoir of allelic variation serves as a strategic resource for sorghum breeding, particularly in the development of climate-resilient cultivars. Therefore, the *Feterita* gene pool is a vital resource for breeding programs targeting traits such as stay-green, drought tolerance, and yield stability in stress-prone environments.

Our study indicates that most of the genetic variation in Sudanese *Feterita* sorghum resides within populations (94%), with only a small proportion (6%) partitioned among populations (P). This pattern suggests that the species exhibits weak regional differentiation and substantial intrapopulation diversity, accompanied by high gene flow (Nm = 6.117). Similar results have been reported in other sorghum collections, where diversity was largely maintained within rather than among geographic groups (Barro-Kondombo et al., 2010; Mengistu et al., 2020).

Our study indicated that pairwise Fst values highlighted West Darfur and Blue Nile as more genetically distinct populations, with moderate to high differentiation (Fst = 0.467–0.528), suggesting restricted gene flow, possibly shaped by geographic barriers or sociopolitical isolation. In contrast, the Kordofan, White Nile, and Al-Gezira populations exhibited low differentiation (Fst = 0.020–0.050), forming a coherent genetic cluster likely facilitated by historical trade routes and shared agricultural networks. The overall weak geographic differentiation across Sudanese *Feterita* reflects traditional seed management systems, where farmer-mediated exchange and informal distribution maintain genetic connectivity and counteract genetic drift (Thomas et al., 2011). These practices ensure that *Feterita* remains a genetically coherent and resilient gene pool, providing valuable diversity for future breeding programs targeting climate adaptation in Sorghum. However, the STRUCTURE results (K = 2) and PCoA collectively indicated that the *Feterita* population represented a genetically continuous but partially structured gene pool. The presence of two ancestral clusters, each shared across multiple regions, likely reflects historical seed diffusion networks rather than recent isolation. Similar weak clustering patterns have been reported in farmer-maintained sorghum landraces from East and West Africa (Barro-Kondombo et al., 2010; Rabbi et al., 2010), where extensive seed exchange maintains allelic diversity within the regions. This admixture underlines the high gene flow (Nm = 6.117) observed and supports the interpretation that *Feterita* genotypes form a single breeding continuum suitable for genetic improvement in the breed.

Our findings revealed that the Sudanese *Feterita* gene pool harbors stay-green alleles of broad agronomic significance, linking drought resilience to yield stability. *SnpSB0054* (SGR1) exhibited a dual role, enhancing plant height while promoting earlier flowering, a drought escape mechanism also highlighted by Borrell et al. (2014). *SnpSB0072* (APETALA2/EREBPs) was correlated with longer panicles, reflecting the role of stress-responsive transcription factors in reproductive development. *SnpSB0101* (SGR3) was strongly associated with grain number and seed yield, consistent with the findings of Mwamahonje et al. (2021). Together, these results tell a compelling story: the alleles that delay senescence and sustain green leaf areas also shape the yield outcomes. Therefore, the Sudanese *Feterita* germplasm is more than just a relic of traditional farming; it is a living genetic archive in which adaptation and productivity are interlinked. Unlocking these alleles through marker-assisted breeding can accelerate the development of drought-resistant sorghum cultivars, offering farmers resilience, stability, and hope in the face of climate change.

## 5 Conclusions

This study provides the first integrated phenotypic and molecular assessment of the Sudanese *Feterita* sorghum gene pool and demonstrates its strong potential as a source of drought-resilient alleles for breeding programs. The wide phenotypic variation observed, particularly in senescence, flowering behavior, and grain yield components, offers clear opportunities for selecting parents suited for post-flowering drought tolerance. Marker–trait associations confirmed the functional relevance of the stay-green loci SGR1 (*snpSB0054*) and SGR3 (*snpSB0101*), highlighting their immediate applicability in marker-assisted selection for improving canopy maintenance and yield stability under terminal drought conditions.

The genetic diversity patterns, characterized by high intrapopulation variation and extensive gene flow, indicate that *Feterita* constitutes a genetically coherent yet allelic-rich resource suitable for allele pyramiding and pre-breeding work. These findings provide a practical foundation for integrating Feterita into breeding pipelines through the targeted introgression of stay-green alleles, development of pre-breeding populations, and future multi-environment testing. Overall, this study positions *Feterita* as a strategically important germplasm pool for climate-smart improvements in sorghum. By enabling the deployment of validated stay-green markers and phenotypically superior accessions, these results support the development of drought-resilient cultivars that respond directly to the production challenges in Sudan’s semi-arid farming systems.

## Data Availability

The datasets presented in this study can be found in online repositories. The names of the repository/repositories and accession number(s) can be found in the article/**Supplementary Material**.
